# Supplementary material for: Vaginal Progesterone Has No Diabetogenic Potential in Twin Pregnancies: A Retrospective Case-Control Study on 1686 Pregnancies
Source: J Clin Med. 2020 Jul 15;9(7):2249. doi: 10.3390/jcm9072249 (PMC7408737; doi:10.3390/jcm9072249)
Supplement: Supplementary file 1 [file jcm-09-02249-s001.pdf]

Supplementary

Table S1. Results of the binary logistic regression model for the prediction of gestational diabetes mellitus development in twin pregnancies with spontaneous conception.

	Women with GDM (n = 205)	Women without GDM (n = 907)	OR (95% CI)	*p*
Vaginal progesterone treatment	17 (8.3)	65 (7.2)	1.339 (0.724;2.474)	0.352
Maternal age (years)	31.9 ± 5.1	30.96 ± 6.29	1.039 (1.007;1.072)	0.017
Pre-pregnancy BMI (kg/m2)	26.5 ± 6.0	23.5 ± 4.6	1.095 (1.062;1.129)	<0.001
Family history of diabetes	69 (33.7)	204 (22.5)	1.274 (0.888;1.828)	0.188
Previous pregnancy with GDM	28 (13.7)	14 (1.5)	7.390 (3.558;15.348)	<0.001
Betamethasone 12mg 2x within 24 hours	69 (33.7)	368 (40.6)	1.449 (0.654;3.212)	0.361
Atosiban* for at least 48 hours	64 (31.2)	357 (39.4)	0.668 (0.296;1.506)	0.331
Current smoking	39 (19.0)	101 (11.1)	1.598(1.021; 2.502)	0.040

BMI: body mass index, OR: odds ratio, 95%CI: 95% confidence interval. Multivariate analyses included all parameters significant in the univariate analysis; *In the atosiban group, women received a bolus injection of 6.75 mg intravenous in 1 min, followed by 18 mg/h. for 3 h, followed by a maintenance dosage of 6 mg/h for 45 h.
